# Impact of Pneumococcal Conjugate Vaccine on Pediatric Tympanostomy Tube Insertion in Partial Immunized Population

**DOI:** 10.1155/2015/248678

**Published:** 2015-03-09

**Authors:** Mao-Che Wang, Ying-Piao Wang, Chia-Huei Chu, Tzong-Yang Tu, An-Suey Shiao, Pesus Chou

**Affiliations:** ^1^Department of Otolaryngology Head and Neck Surgery, Taipei Veterans General Hospital, Taipei, Taiwan; ^2^School of Medicine, National Yang-Ming University, Taipei, Taiwan; ^3^Institute of Public Health and Community Medicine Research Center, National Yang-Ming University, Taipei, Taiwan; ^4^Department of Otolaryngology Head and Neck Surgery, Mackay Memorial Hospital, Taipei, Taiwan; ^5^Department of Audiology and Speech Language Pathology and School of Medicine, Mackay Medical College, New Taipei City, Taiwan

## Abstract

*Objective*. To investigate the impact of seven-valent pneumococcal conjugate vaccine on tube insertions in a partial immunized pediatric population. *Study Design*. Retrospective ecological study. *Methods*. This study used Taiwan National Health Insurance Research Database for the period 2000–2009. Every child under 17 years old who received tubes during this 10-year period was identified and analyzed. The tube insertion rates in different age groups and the risk to receive tubes in different birth cohorts before and after the release of the vaccine in 2005 were compared. *Results*. The tube insertion rates for children under 17 years of age ranged from 21.6 to 31.9 for 100,000 persons/year. The tube insertion rate of children under 2 years old decreased significantly after 2005 in period effect analysis (*β* = −0.074, *P* < 0.05, and the negative *β* value means a downward trend) and increased in children 2 to 9 years old throughout the study period (positive *β* values which mean upward trends, *P* < 0.05). The rate of tube insertion was lower in 2004-2005 and 2006-2007 birth cohorts than that of 2002-2003 birth cohort (RR = 0.90 and 0.21, 95% CI 0.83–0.97 and 0.19–0.23, resp.). *Conclusion*. The seven-valent pneumococcal conjugate vaccine may reduce the risk of tube insertion for children of later birth cohorts. The vaccine may have the protective effect on tube insertions in a partial immunized pediatric population.

## 1. Introduction

Acute otitis media (AOM) is a very common otologic problem in children. Sixty percent of infants experience AOM by the age of 18 months [1]. AOM usually comes after a viral upper respiratory tract infection. Symptoms and signs are the acute onset of otalgia, fever, hearing deterioration, nausea, vomiting, and otorrhea, if tympanic membrane ruptures.* Streptococcus pneumoniae* is the most frequently identified pathogen, which accounts for 25 to 50% of AOM, followed by* Haemophilus influenzae* and* Moraxella catarrhalis*, which are responsible of 15 to 30% and 3 to 20% of AOM episodes, respectively [2]. AOM can resolve spontaneously or after treatment. The condition can also develop into otitis media with effusion (OME) or recurrent AOM. Ninety percent of children have an episode of OME before school age, especially from the age of 6 months to 4 years [3, 4]. Most OME resolves within three months, but 30–40% of patients may have recurrent OME [3, 5, 6]. Observation and medical treatment are conservative management strategies for AOM and OME. Tympanostomy tube insertion is the preferred surgical procedure for these conditions. The American Academy of Otolaryngology-Head and Neck Surgery (AAO-HNS) set the clinical practice guidelines for OME in 2004 and clinical practice guidelines for tympanostomy tubes in children in 2013. The guidelines recommended that clinicians offer bilateral tympanostomy tubes to children with bilateral chronic OME (OME lasting for 3 months or longer) and recurrent AOM with middle ear effusion. The guidelines also recommended that clinicians should not perform tympanostomy tubes to children with a single episode of OME lasting less than 3 months or recurrent AOM without middle ear effusion [7, 8]. Tympanostomy tube insertion is the treatment of choice for pediatric recurrent acute otitis media and chronic otitis media with effusion.

The seven-valent pneumococcal conjugate vaccine (PCV7) was released in the United States in the year 2000. This vaccine was used to prevent pediatric pneumococcal disease. After immunization with PCV7 had been ongoing for more than a decade all over the world, it was demonstrated to be very effective in reducing mortality related to pneumococcal disease, invasive pneumococcal disease (IPD), pneumonia, and otitis media in children [9–15]. Immunization with PCV7 also reduced penicillin nonsusceptible strains of pneumococcus and vaccine type pneumococcus colonization in the nasopharynx. Serotype changes in IPD pathogens and nasopharyngeal carriage were also noted [16–27]. The protective effect against pneumococcal disease of PCV7 vaccination was found not only for vaccinated children, but also for unvaccinated children and adults. Many studies found that IPD and pneumonia of adults decreased significantly after increased coverage rate of PCV7 vaccination in infants and children, which showed there were both direct and indirect (herd) effects of the vaccine [10, 17, 28–35]. In addition to protective effects against pneumonia and IPD, PCV7 also reduced pediatric otitis media and the rate of tympanostomy tube insertions. In comparison with pneumonia and IPD, otitis media affects more children and it is associated with a greater social and economic burden than any other pneumococcal diseases [36–38]. During the past decades, numerous studies have tried to identify the protective effect of PCV7 on pediatric otitis media. Some studies were randomized controlled trials [39–46], some were analyses of secondary data [9, 47–53], and others were systemic reviews [11, 54–59]. Most studies showed PCV7 did have the protective effect on pediatric otitis media in the perspectives of reducing AOM episodes, AOM visits, physician claims for otitis media, antibiotic prescriptions, and tympanostomy tube insertion rates [9, 11, 39–53, 57–59]. However, some studies of the vaccine showed negative results [54–56]. These studies all focused on children vaccinated with PCV7.

PCV7 was first introduced to Taiwan in the year 2005. It was not included into the national immunization program and was only free for high-risk children such as children with rare disease or malignancy, or parents had to pay USD 110 for each dose of the vaccine from their own pocket. We conducted this ecological study to examine the impact of PCV7 on reducing the rate of pediatric tympanostomy tube insertions, which was a surrogate of complicated and intractable pediatric otitis media in a partial immunized pediatric population.

## 2. Materials and Methods

This ten-year study (2000–2009) used Taiwan National Health Insurance Research Database (NHIRD), population-based data on approximately 99% of the 23 million legal residents covered by the National Health Insurance in Taiwan. Every admission and outpatient visit record was included in this database. National Health Insurance is the only buyer of medical service in Taiwan and NHIRD is released for academic use yearly by the National Health Institute of Taiwan. This study was reviewed and approved by the institutional review board of the Taipei Veterans General Hospital. All children who had tympanostomy tube insertion before they reached 17 years of age (2000 to 2009) were included in this study. The study population was obtained by retrieving all of the patients with the procedure codes for myringotomy with ventilation tube insertion under a microscope, from the claims data of the NHIRD, with ages under 17 years on the date of the surgery. This was a population-based data without any sampling. We stratified the children into four age groups (0 ≦ age < 2, 2 ≦ age < 5, 5 ≦ age < 9, and 9 ≦ age < 17). We examined the rate of tympanostomy tube insertion in each age group in each calendar year during the study period from 2000 to 2009. We also compared the rate of tympanostomy tube insertion of children in 2002-2003, 2004-2005, and 2006-2007 birth cohorts.

## 3. Statistical Analysis

Age and calendar year-specific rates of pediatric tympanostomy tube insertion were calculated as the number of children who had undergone tympanostomy tube insertion divided by the total registered population per 100000 in the given stratum of age and year. The registered population was obtained from the Taiwan National Statistics Report [60]. A graphical approach was used to describe the age-specific rates of tympanostomy tube insertion over calendar years and to describe the rates across ages by birth cohorts.

We used the Poisson regression method to examine time trend of rates of tympanostomy tube insertion. Given that pneumococcal conjugate vaccine was introduced and has been available since 2005 in Taiwan, we assumed that the period effects could have varied before and after 2005. As a result, a segmented regression analysis, also known as piecewise regression, was used to identify intervention effects of vaccine being introduced in Taiwan on the rates of tympanostomy tube insertion [61]. Period effects were separated into two segments, one for trend before 2005 and the other after 2005, and thus the time trend of rates of tympanostomy tube insertion can be estimated by two regression parameters.

A multiphase method was used to examine possible cohort effects [62]. This approach considers cohort effects as the systemic component of multiplicative interaction between age and calendar year. The first step is to examine whether cohort effect exists by using graph of age-specific rate of tympanostomy tube insertion across years created from age-period contingency table. Next, median polish analyses for log-transforming rates were performed. The residuals generated from the previous step were used to estimate the relative magnitude of cohort effects using linear regression. The exponentiated regression coefficients indicate the excess rate attributable to each cohort category.

## 4. Results

**Figure 1:**
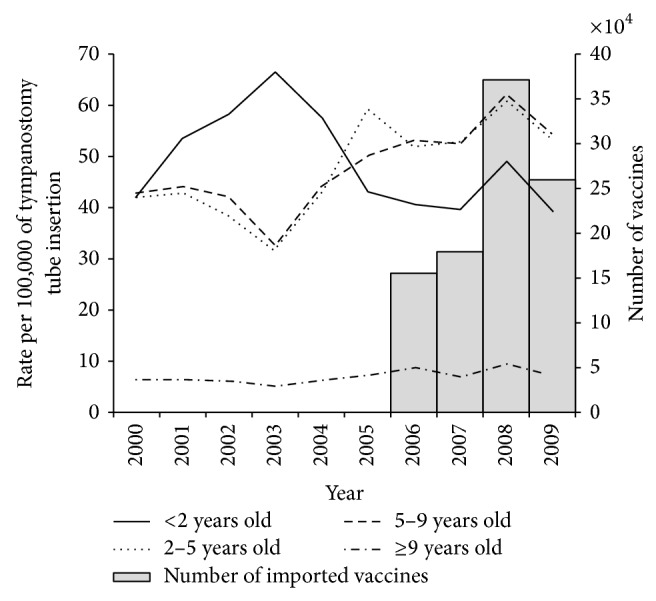
Age-specific rate of tympanostomy tube insertion from 2000 to 2009 and number of imported pneumococcal conjugate vaccines from 2006 to 2009.

**Figure 2:**
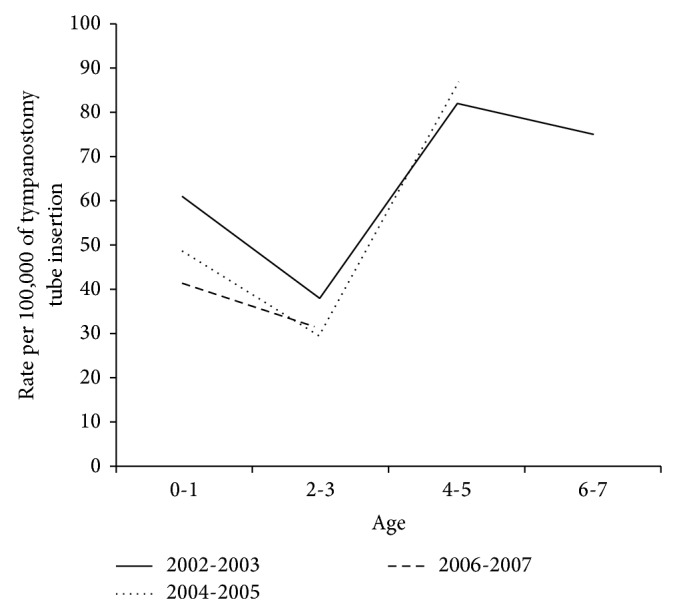
Birth cohort-specific rate of tympanostomy tube insertion across ages.

From the year 2000 to 2009, 13,074 patients receiving tympanostomy tube insertion under 17 years of age were included into this study. The patient numbers and tympanostomy tube insertion rates in each age stratum and in each calendar year were showed in Table 1. The tube insertion rates for children under 17 years of age ranged from 21.6 to 31.9 for 100,000 persons/year. The tympanostomy tube insertion rate of children showed an overall increasing trend with time except for children under 2 years old, which had a decreasing trend after 2005. There was a surge of increased rates in 2008 for children under 9 years old and then a decrease in 2009, which may have been the effect of the increased availability of imported vaccine in 2008 (Figure 1). The tympanostomy tube insertion rate for children less than 2 years old decreased significantly after 2005 (*β* = −0.074, *P* < 0.05, and the negative *β* value means a downward trend) and increased in children 2 to 9 years of age throughout the study period (positive *β* values which mean upward trends, *P* < 0.05) (Table 2). However, the tympanostomy tube insertion rate for children less than 2 years old seemed to be decreasing since 2003 before the introduction of the PCV7 in 2005 (Figure 1). The tympanostomy tube insertion rates of birth cohorts 2004-2005 and 2006-2007 were significantly lower than that of the birth cohort 2002-2003 (RR = 0.90 and 0.21, 95% CI 0.83–0.97 and 0.19 = 0.23, resp.). The risk of tube insertion of the birth cohort 2006-2007 after the introduction of the PCV7 was only one-fifth that of the birth cohort 2002-2003 before the introduction of the vaccine in Taiwan (Figure 2 and Table 3).

## 5. Discussion

Otitis media is a very common and important otologic problem in children. This condition is one of the most frequent causes of clinic visits and antibiotics prescriptions in children [36–38]. The surgical management of complicated and intractable otitis media, involving tympanostomy tube insertions, is one of the most frequent surgical procedures for children requiring general anesthesia [63]. Thus the prevention of pediatric otitis media is a very important issue. The most common pathogen of pediatric otitis media is* Streptococcus pneumonia*, which accounts for 30% to 50% of the pathogens associated with pediatric acute otitis media [2]. PCV7 can only offer partial protection against pediatric otitis media. We can see the protective effect of PCV7 on tube insertion in this study which meant the magnitude of reduction on tube insertion caused by pneumococcus would be even more significant. PCV7 was not initially included into national immunization program in Taiwan. According to the data from Taiwan Center of Disease Control, there were 962707 doses of PCV7 imported to Taiwan by the end of the year 2009. There were 798916 babies born in Taiwan from 2006 to 2009 and there were 1956143 children under 9 years of age in Taiwan in 2009 [60]. Newborn infants after 2005 could receive 1 to 4 doses of the vaccine and children who were 2–9 years old after 2006 could receive 1 dose of the vaccine. It is estimated that there could be up to 30.13% newborn infants after 2005 who were vaccinated or at most 49.2% of children under 9 years old who were immunized in 2009. To the best of our knowledge, this study is the first that has attempted to uncover the protective effect of PCV7 on pediatric otitis media or tympanostomy tube insertion in a partial vaccinated pediatric population. In this study, we found the decreased tympanostomy tube insertion rate in children younger than 2 years of age after the introduction of PCV7 in 2005 by period effects; however we also found that the decreased tympanostomy tube rate seemed to be started in 2003. As a result we could not conclude that the decreased tympanostomy tube rate was the protective effect Of PCV7. For children over 2 years of age, the tympanostomy tube insertion rate increased through the study period from 2000 to 2009 which meant we could not see the protective effect of PCV7 on tympanostomy tube insertions for children older than 2 years of age. However, we also found the tympanostomy tube insertion rate decreased significantly to one-fifth in the birth cohort (2006-2007 birth cohort) after introduction of the vaccine in comparison with the birth cohort (2002-2003 birth cohort) before the vaccine's introduction. This implied that there was a protective effect of PCV7 on tympanostomy tube insertion rates in this partially vaccinated population in Taiwan. In the analysis of cohort effect, we found children of 2004-2005 and 2006-2007 birth cohort had lower rates of tympanostomy tube insertion than that of children of 2002-2003 birth cohort before the age of 3. The difference of rates of tympanostomy tube insertion in children between 2002-2003 and 2004-2005 birth cohorts disappeared when the age reached 4 to 5. (Figure 2) The reason of these results we found may be that no PCV7 was available in Taiwan when children of 2002-2003 were younger than 3 years old and none of them was vaccinated. When children of 2002-2003 birth cohort reached 4 years old, PCV7 was introduced to Taiwan and some of them were vaccinated and became immunized leading to similar tympanostomy tube insertion rate with children of 2004-2005 birth cohort. This further supported the protective effect of PCV7 on tympanostomy tube insertion in this partial immunized pediatric population in Taiwan.

In this study, we found an overall increased trend of tympanostomy tube insertion rates in the study period from 2000 to 2009 in Taiwan (except for children under 2 years old after the introduction of PCV7 in 2005). Other studies have reported a downward trend of tympanostomy tube insertion in the United States from 1995 to 2005 [37, 47, 49, 50, 52, 59]. The increased trend in tube insertion rates in Taiwan may have been due to the progressive improvement accessibility of medical service since the beginning of National Health Insurance in 1995. Other factors such as better training of clinicians and diagnostic instruments have continuously been improving in Taiwan over the past decade. One other possible explanation of increased trend of tube insertion even after introduction of PCV7 is serotype replacement of pneumococcus. According to the report of Taiwan Centers for Disease Control, IPD in children under 5 years old caused by serotype 19A increased from 12.2% in 2008 to 43.4% in 2011. The increase of this multidrug resistant strain may also lead to the increase of intractable pediatric otitis media. Despite the increasing trend of tympanostomy tube insertion in Taiwan, we have a lower rate of tympanostomy tube insertion in comparison with that in the United States and Europe [64–66]. The reasons may be that surgical interventions are not favorable choices of Taiwanese parents, leading to more conservative management, or otolaryngologists in Taiwan adhered more to the suggestions of clinical practice guideline in comparison to surgeons in the United States on treatment of pediatric otitis media with effusion [67–69]. For previous studies analyzing observational database, several of them also found protective effect PCV on otitis media for children [9, 37, 47, 48]. Poehling et al. demonstrated less frequent otitis media visits and tube insertions in a more recent cohort of children covered by Tenn Care and New York private insurance [48]. However, we found those similar protective effects in a partially immunized pediatric population without a universal immunization program for PCV7.

To improve the internal validity of this study, tympanostomy tube insertion is used instead of diagnosis codes in ICD-9 as a surrogate of chronic OME and recurrent AOM for the accuracy of defining the study population. If there was a code for certain surgical procedures for a particular patient in the claims data, that patient was considered to have the disease and therefore underwent a surgical procedure to address it. In contrast, if diagnosis codes in ICD-9 were used as a surrogate for the disease, the probability of miscoding by the physician could have been much higher. Physicians may use the wrong diagnosis code during instances of misdiagnoses. They also may do this for prescribing antibiotics or laboratory test in order to pass the review of the insurance payer or to improve reimbursement.

In this study we used a population-based database, NHIRD, without sampling for the analysis. However, the major limitation of this study is the limitation of the administrative claims data. Medical records and the operative notes of every patient could not be obtained. Another limitation is that, at the beginning of PCV7 vaccination in Taiwan, PCV7 was only for select patients. PCV was not included in national universal vaccination program until 2013. We could not identify those vaccinated children in the NHIRD in Taiwan. Therefore, we were unable to examine the difference of tympanostomy insertion rates between vaccinated and unvaccinated children, which means we could not determine if there was a direct effect of PCV7 on tympanostomy tube insertion rates. For this reason, we could not distinguish whether the protective effect was due to a direct effect or indirect (herd) effect. We can only make the inference that the protective effect was a direct effect or combination of both direct and indirect effects. There are other limitations of this study. There is surveillance bias in the study. Parents who are willing to pay for the vaccine may care more about the health condition of the children and may seek for medical care more. The vaccinated children may be easier to be picked up to have middle ear condition than unvaccinated children. Fortunately, this surveillance bias leads to underestimation of the protective effect of the vaccine which may not change the direction of the result of this study. In this study we use the imported vaccine number as the surrogate of total vaccine delivered to the children. This must be an overestimation of the vaccination which also leads to underestimation of the protective effect of the vaccine.

## 6. Conclusion

Tympanostomy tube insertion rates in birth cohorts after 2005 were lower than those in the birth cohort before 2005. PCV7 may have protective effect on pediatric tympanostomy tube insertion rates in a partially immunized population in Taiwan.

## Figures and Tables

**Table 1:** The case number and rate of tympanostomy tube insertion in each age stratum and each calendar year.

Age	2000		2001		2002		2003		2004		2005		2006		2007		2008		2009	
	*n*	Rate^a^	*n*	Rate^a^	*n*	Rate^a^	*n*	Rate^a^	*n*	Rate^a^	*n*	Rate^a^	*n*	Rate^a^	*n*	Rate^a^	*n*	Rate^a^	*n*	Rate^a^
Total	1347	24.9	1406	26.5	1321	25.4	1109	21.6	1287	25.5	1389	28.3	1338	27.9	1253	26.8	1453	31.9	1171	26.5
0	106	36.2	143	58.0	116	49.0	140	64.4	121	58.5	71	36.3	86	44.6	76	39.6	115	61.3	90	49.3
1	136	48.1	153	50.0	172	66.9	168	68.2	129	56.7	107	49.0	77	37.0	82	39.7	78	38.0	60	30.2
2	76	28.4	42	14.8	59	19.2	52	20.2	50	20.3	63	27.7	42	19.2	34	16.3	60	29.0	51	24.8
3	110	34.0	117	43.7	80	28.3	59	19.3	96	37.4	117	47.5	98	43.1	97	44.4	94	45.2	68	32.9
4	199	61.6	216	66.7	190	71.0	156	55.2	202	66.0	253	98.4	218	88.5	214	94.1	230	105.3	211	101.3
5	216	66.5	234	72.4	216	66.7	166	62.0	222	78.5	237	77.3	249	96.8	247	100.2	261	114.7	203	92.8
6	178	55.1	184	56.6	165	51.1	119	36.8	163	60.9	179	63.2	174	56.8	181	70.4	200	81.1	152	66.7
7	98	30.0	95	29.4	99	30.5	77	23.9	79	24.4	95	35.5	98	34.6	80	26.1	110	42.8	97	39.4
8	62	19.3	59	18.0	64	19.8	40	12.3	63	19.5	81	25.0	71	26.6	66	23.3	71	23.2	64	24.9
9	44	13.8	47	14.6	45	13.8	45	14.0	42	12.9	59	18.3	65	20.1	43	16.1	69	24.4	44	14.4
10	32	9.5	44	13.8	22	6.9	27	8.3	40	12.4	30	9.2	47	14.6	44	13.6	49	18.3	35	12.4
11	37	11.9	23	6.9	36	11.3	16	5.0	24	7.4	41	12.7	48	14.8	34	10.5	49	15.2	22	8.2
12	21	6.1	25	8.0	27	8.1	14	4.4	16	5.0	25	7.7	33	10.2	12	3.7	26	8.1	37	11.4
13	17	5.5	8	2.3	11	3.5	12	3.6	16	5.0	13	4.1	17	5.2	15	4.7	15	4.6	14	4.3
14	7	2.3	8	2.6	7	2.1	12	3.9	10	3.0	9	2.8	4	1.2	14	4.3	12	3.7	11	3.4
15	3	0.9	5	1.7	7	2.3	3	0.9	11	3.5	4	1.2	6	1.9	6	1.9	9	2.8	4	1.2
16	5	1.4	3	0.9	5	1.7	3	1.0	3	0.9	5	1.6	5	1.5	8	2.5	5	1.6	8	2.5

^a^Incidence rate per 100,000.

**Table 2:** Estimates of piecewise terms in Poisson regression models for evaluation of effects of pneumococcal conjugate vaccines against pneumococcal infection on tympanostomy tube insertion among children.

	0–2 years old		2–5 years old		5–9 years old		9–17 years old	
	*β*	se	*β*	se	*β*	se	*β*	se
Before 2005	0.004	0.014	0.052^*^	0.012	0.029^*^	0.009	0.037^*^	0.017
After 2005	−0.074^*^	0.021	0.041^*^	0.015	0.064^*^	0.012	0.039	0.020

^*^ *P* < 0.05.

**Table 3:** Estimated rate ratios and 95% confidence intervals for the effect of birth cohort on tympanostomy tube insertion rate per population of 100,000.

	RR	95% CI
2002-2003 cohort	1.00	
2004-2005 cohort	0.90	0.83–0.97^*^
2006-2007 cohort	0.21	0.19–0.23^*^

RR: rate ratios.

CI: confidence interval.

## Abbreviations

AAO-HNS: American Academy of Otolaryngology-Head and Neck Surgery
AOM: Acute otitis media
CI: Confidence interval
IPD: Invasive pneumococcal disease
NHIRD: National Health Insurance Research Database
PCV7: Seven-valent pneumococcal conjugate vaccine
OME: Otitis media with effusion
RR: Rate ratio.
